# Engineering rice with lower grain arsenic

**DOI:** 10.1111/pbi.12905

**Published:** 2018-03-25

**Authors:** Fenglin Deng, Naoki Yamaji, Jian Feng Ma, Sang‐Kyu Lee, Jong‐Seong Jeon, Enrico Martinoia, Youngsook Lee, Won‐Yong Song

**Affiliations:** ^1^ Department of Integrative Bioscience and Biotechnology Pohang University of Science and Technology Pohang Korea; ^2^ Institute of Plant Science and Resources Okayama University Kurashiki Japan; ^3^ Graduate School of Biotechnology & Crop Biotech Institute Kyung Hee University Yongin Korea; ^4^ Institute of Plant Biology University Zurich Zurich Switzerland

**Keywords:** rice, arsenic, vacuolar sequestration, ABC transporter

## Abstract

Arsenic (As) is a poisonous element that causes severe skin lesions and cancer in humans. Rice (*Oryza sativa* L.) is a major dietary source of As in humans who consume this cereal as a staple food. We hypothesized that increasing As vacuolar sequestration would inhibit its translocation into the grain and reduce the amount of As entering the food chain. We developed transgenic rice plants expressing two different vacuolar As sequestration genes, *ScYCF1* and *OsABCC1*, under the control of the RCc3 promoter in the root cortical and internode phloem cells, along with a bacterial γ‐glutamylcysteine synthetase driven by the maize UBI promoter. The transgenic rice plants exhibited reduced root‐to‐shoot and internode‐to‐grain As translocation, resulting in a 70% reduction in As accumulation in the brown rice without jeopardizing agronomic traits. This technology could be used to reduce As intake, particularly in populations of South East Asia suffering from As toxicity and thereby improve human health.

## Introduction

The US Agency for Toxic Substances and Disease Registry (ATSDR) ranked arsenic (As) top among hazardous substances in their 2015 Priority List (http://www.atsdr.cdc.gov/spl). As is widely dispersed around the world; in addition to being a natural component of Earth's lithosphere, As is released into the environment through anthropogenic pollution (Bowell *et al*., 2014). Rice (*Oryza sativa* L.) is a major contributor to inorganic As intake for the global population, especially for Asians, who consume rice as a staple food (Meharg, 2004; Meharg *et al*., 2009; Schoof *et al*., 1999; Williams *et al*., 2007; Zhao *et al*., 2010). Rice cultivated in paddy fields accumulates arsenite and arsenate in its grain through the same uptake and translocation pathways used to acquire silicon and phosphate, respectively. As silicon and phosphate are essential macroelements for rice (Li *et al*., 2016; Ma *et al*., 2008; Zhao *et al*., 2010). Efforts to reduce As intake cannot be based on blocking these pathways but should rather focus on removing As from the environment, including agricultural fields, or reducing As accumulation in the edible parts of the plant (i.e. grain). Removing As from the environment to an extent that would benefit human health is not feasible, because As is widespread and large tracts of agricultural land are contaminated with As (Bowell *et al*., 2014). Several strategies have been proposed to reduce As accumulation in rice grains, including breeding rice cultivars with low As accumulation, applying silicon fertilizers to limit arsenite uptake and reducing As levels in paddy soil by water management (Arao *et al*., 2009; Sun *et al*., 2014; Wang *et al*., 2016a; Zhao *et al*., 2010). However, these strategies have limitations. The application of Si fertilizer reduced grain As content by only 20% (Wang *et al*., 2016a). In addition, water management for aerobic cultivation caused cadmium accumulation in rice grain and reduced rice yield (Arao *et al*., 2009; Sun *et al*., 2014). Therefore, developing crops with reduced As levels in their edible parts is the most plausible strategy for decreasing human As intake.

Many studies have reported the mechanisms and genes involved in As uptake, translocation, accumulation and resistance (Punshon *et al*., 2017; Shi *et al*., 2016; Wang *et al*., 2016b; Xu *et al*., 2017; Yang *et al*., 2016), but few have examined how rice plants reduce the As content in their grain (Ma *et al*., 2008; Song *et al*., 2014). For example, loss‐of‐function rice plants lacking the arsenic efflux transporter Lsi2 showed a reduced As content in the grain (51%–63% that of the wild type (WT)) but also a reduced grain yield (only 40% that of the WT; Ma *et al*., 2007, 2008). An alternative approach is therefore needed to decrease the As contents of the grain without impairing rice yield.

Vacuolar sequestration has been suggested as a crucial mechanism for the resistance, accumulation and differential distribution of toxic metal(loid)s in plants. The expression of *Saccharomyces cerevisiae yeast cadmium factor* (*ScYCF1*), a vacuolar glutathione‐heavy metal(loid) conjugate, enhanced cadmium (Cd) and lead (Pb) resistance and accumulation in *Saccharomyces cerevisiae* and in plants (Shim *et al*., 2013; Song *et al*., 2003). In addition, *ScYCF1* was found to transport bis‐glutathione‐As and contribute to As detoxification in yeast (Ghosh *et al*., 1999). By contrast, a double knockout mutant lacking the two vacuolar ABC transporters that constitute the major phytochelatin‐arsenic complex transporters in Arabidopsis, *AtABCC1* and *AtABCC2*, exhibited a strong As hypersensitive phenotype (Song *et al*., 2010). *OsABCC1,* the closest homologue of AtABCC1 and AtABCC2, was found to be important for reducing the As concentration in rice (Song *et al*., 2014); the grains of the *osabcc1* knockout lines accumulated 10 times more As than the WT. OsABCC1 is mainly located in the vacuolar membrane of the nodal phloem companion cells. Together, these results indicated that *OsABCC1*, expressed in specific cells of the nodes, sequesters As into the vacuole, blocking As translocation to the grains (Song *et al*., 2014). Overexpression of *heavy metal ATPase* (*HMA3*), a heavy metal ATPase regulating Cd sequestration into the vacuole, reduced Cd translocation to the rice shoot, including the grains (Ueno *et al*., 2010).

In this study, we developed transgenic rice exhibiting reduced As concentration in grain with the strategy of vacuolar As sequestration into cortex and phloem cells of root and internode to inhibit As translocation into grains.

## Results and discussion

### Development of transgenic rice with *OsABCC1*, γ*‐ECS* and *ScYCF1*

**Figure 1:**
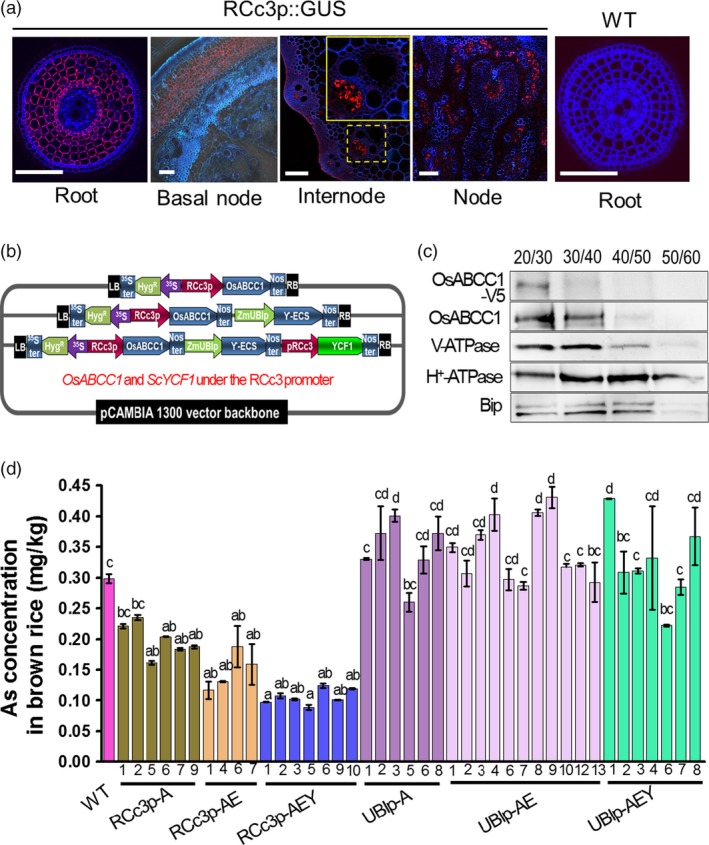
Development of transgenic rice with reduced As accumulation in the grains using tissue‐specific expression of *OsABCC1*, γ*‐ECS* and *ScYCF1*. (a) Analysis of the tissue‐specific expression of the *OsRCc3* promoter by immunostaining *OsRCc3* promoter::GUS transgenic plants (*RCc3p::GUS*) using a GUS antibody. The internode section denoted by broken lines is magnified in the inset surrounded by yellow solid lines. Bar = 100 μm. (b) Maps of vectors carrying *RCc3*p‐A (*RCc3* pro::*OsABCC1‐V5*), RCc3p‐AE (*RCc3* pro::*OsABCC1‐V5, ZmUBI* pro::γ*‐ECS*) or RCc3p‐AEY (*RCc3* pro::*OsABCC1‐V5, ZmUBI* pro::γ*‐ECS, RCc3* pro::*ScYCF1*). (c) Subcellular localization of OsABCC1‐V5 in the roots of RCc3p‐AEY plants using a sucrose density gradient analysis. Anti‐V5, anti‐OsABCC1, anti‐V‐ATPase (tonoplast marker), anti‐H^+^‐ATPase (plasma membrane marker) and Bip (ER marker) were used to assess protein abundance in the different compartments. The signal of OsABCC1‐V5 corresponded to the signals of OsABCC1 and V‐ATPase. (d) Arsenic accumulation in brown rice harvested from T3 transgenic plants transformed with RCc3p‐A, RCc3p‐AE, RCc3p‐AEY, UBIp‐A, UBIp‐AE or UBIp‐AEY. The As concentration was analysed in brown rice from plants grown in soil with a typical basal level of As. The values are means and standard errors (*n* = 5 plants). Different letters indicate significantly different means (Tukey's multiple comparison analysis, *P* ≤ 0.05).

To reduce As accumulation in rice grains, we developed transgenic rice expressing two different vacuolar As sequestration genes, *ScYCF1* and *OsABCC1*. These genes were expressed concomitantly with a bacterial γ‐glutamylcysteine synthetase (γ*‐ECS*), a key enzyme in glutathione synthesis, to increase the nonprotein thiol (glutathione and phytochelatin) concentration in the cytosol (Ghosh *et al*., 1999; Song *et al*., 2014; Zhu *et al*., 1999). We produced rice lines expressing either V5 tag fused *OsABCC1* alone (*OsABCC1‐V5,* A for short in constructs), *OsABCC1‐V5* together with γ*‐ECS* (AE) or *OsABCC1‐V5*, γ*‐ECS* and *ScYCF1* (AEY). *OsABCC1* and *ScYCF1* were driven by the rice *root‐specific cDNA clone3* (*RCc3*) promoter for root cortical cell‐, internode‐ and node‐specific promoter expression (Xu *et al*., 1995; Figures S1 and 1a), and γ*‐ECS* was constitutively expressed under the *Zea mays ubiquitin* (*ZmUBI*) promoter, a ubiquitous promoter (Figure S2). The corresponding constructs were named RCc3p‐A (*RCc3* promoter::*OsABCC1‐V5*), RCc3p‐AE (*RCc3* promoter::*OsABCC1‐V5*,* ZmUBI* promoter::γ*‐ECS*) and RCc3p‐AEY (*RCc3* promoter::*OsABCC1‐V5*,* ZmUBI* promoter::γ*‐ECS*,* RCc3* promoter::*ScYCF1*) (Figure 1b). In addition, to determine whether the cell‐specific expression of vacuolar As transporters in the root cortex or the phloem of the node and internode is critical for reducing As accumulation in grains, we prepared the following control transgenic plants expressing *OsABCC1* and *ScYCF1* using a *ZmUBI* promoter: UBIp‐A (*ZmUBI* promoter::*OsABCC1‐V5*), UBIp‐AE *(ZmUBI* promoter::*OsABCC1‐V5*,* OsActin2* promoter::γ*‐ECS*) and UBIp‐AEY (*ZmUBI* promoter::*OsABCC1‐V5*,* OsActin2* promoter::γ*‐ECS*,* ZmUBI* promoter::*ScYCF1*) (Figure S4a). We first confirmed that all the expression cassettes were successfully inserted into the rice genome and expressed in the transgenic plants (Figures S3a, b, c and S4b, c). We also confirmed that OsABCC1‐V5 protein was produced in the RCc3p‐AEY and UBIp‐AEY lines (Figures S3d and S4d) and that the fused protein was successfully targeted to the vacuolar membrane of the roots of the RCc3p‐AEY plants (Figure 1c).

### Reduced As accumulation in grains from transgenic rice plants expressing *OsABCC1* and *ScYCF1* under the *RCc3* promoter

As concentrations in brown rice were analysed from T3 transgenic rice plants cultivated in normal soil with a typical basal level of As (2.8 ± 0.5 mg/kg dried soil; below the acceptable soil As limits in Korea (25 mg/kg)). As accumulation was reduced in the transgenic lines, including RCc3p‐A, RCc3p‐AE and RCc3p‐AEY. In particular, the brown rice of all RCc3p‐AEY transgenic plants accumulated just 30% of the As levels in WT brown rice (Figure 1d), and As concentrations in the flag leaves of the transgenic lines were highly decreased (Figure S5c). In contrast to the transgenic plants expressing vacuolar As transporters driven by the *RCc3* promoter, all single, double and triple overexpressors of *OsABCC1*, γ*‐ECS* and *ScYCF1* driven by the ubiquitous promoters *ZmUBIp* and *Actin2p* had increased or similar levels of As in their brown rice as the WT (Figure 1d). These results indicate that the tissue‐specific expression of the vacuolar transporters had a greater impact on reduced As levels than did their total expression levels. Interestingly, the Cd concentrations in the grains and flag leaves of RCc3p‐AEY were comparable between the WT and transgenic lines (Figure S5a, b).

### Reduced As translocation from root to the grains in RCc3p‐AEY plants

**Figure 2:**
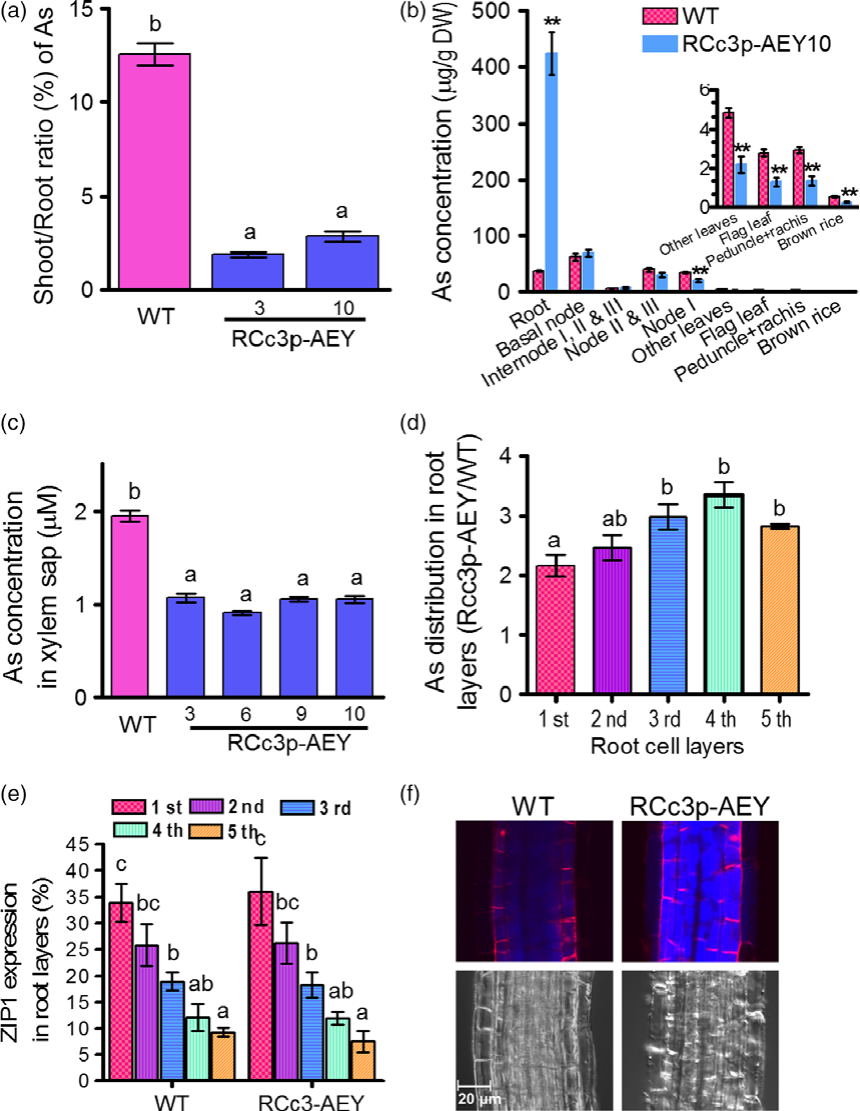
Reduced As translocation into the grains of RCc3p‐AEY plants. (a) Shoot to root As ratio. As contents were measured in the roots and shoots of 4‐week‐old plants treated with 1 μm As(III) for five days, and their shoot to root As ratio was analysed (*n* = 8 plants). (b) As concentrations of various tissues from four‐month‐old mature rice plants treated with 1 μm As(III) for 10 days. All values are means with corresponding standard errors (*n* = 5 plants). *P ≤ 0.05, **P ≤ 0.01 (Student's *t*‐test). (c) As concentration in xylem sap from 4‐week‐old plants treated with 5 μm As(III) for 4 h (*n* = 8 plants). (d) As distribution in different root layers of RCc3p‐AEY plants. As concentrations were measured in successive layers of root samples. The As concentrations were normalized by total protein content and expressed as the ratios of concentrations in RCc3p‐AEY compared with that in WT (*n* = 4 batches of 300 roots). (e) Expression pattern of the epidermis marker gene *OsZIP1* in the successive layers of root samples analysed in d (*n* = s4 batches of 300 roots). The result indicates that the first root cell layer corresponds to the epidermis, the second is a mixture of the epidermis and cortex, the third and fourth are derived from the cortex, and the fifth layer is vascular tissue. (f) Accumulation of thiols in roots of 3‐week‐old plants treated with 1 μm As(III) for 3 h. The thiol signals were obtained by staining with 15 μm monobromobimane for 30 min. The red fluorescence indicates the cell wall stained with 20 μm propidium iodide. The values are means and standard errors. In b, c, d and e, the different letters indicate significantly different means (Tukey's multiple comparison analysis, *P* ≤ 0.05).

All lines exhibited reduced As accumulation in grains and flag leaves compared to the WT and transgenic plants harbouring the UBI‐driven constructs, UBIp‐A, UBIp‐AE and UBIp‐AEY (Figure 1d and Figure S5c). We randomly selected 3–8 transgenic lines to further investigate the mechanisms of As reduction in the brown rice of the RCc3p‐AEY lines. First, we analysed As accumulation in roots and shoots from 4‐week‐old plants treated with 1 μm As(III) for 5 days. The As levels in the roots and shoots of the transgenic rice plants expressing *OsABCC1*, γ*‐ECS* and *ScYCF1* under the ubiquitous promoters did not differ from those of the WT (Figure S6a, b), consistent with the unaffected As levels in the grains (Figure 1d). By contrast, all the RCc3p‐AE (2 lines) and RCc3p‐AEY (2 lines) transgenic plants consistently exhibited different As levels compared to the WT. All of these lines accumulated more than twofold higher As in their roots, but exhibited strongly reduced As levels in their shoots compared to the WT (Figure S6a, b), indicating that the RCc3p‐AEY plants had reduced root‐to‐shoot As translocation (Figure 2a). We then compared the As distribution in various tissues of RCc3p‐9 transgenic and WT plants at the grain‐filling and vegetative stages. During the grain‐filling stage, the RCc3p‐AEY transgenic plants accumulated 10 times more As in the root than the WT, although the levels of As in the basal tissues of the shoot, such as the basal nodes, internodes and nodes II and III, were similar to those of the WT (Figure 2b). Less As was accumulated in the upper parts of the shoot of these transgenic plants than in the WT, including at node I, the leaves, internode I, the rachis and the brown rice (Figure 2b). To determine whether this was indeed the cause of the As retention in the roots of RCc3p‐AEY plants, we analysed the As concentrations in the xylem sap of rice seedlings treated with As (III) for four hours. Consistent with their As distribution, the xylem sap of the RCc3p‐AEY lines had a lower As concentration than the WT (Figure 2c). However, when treated with organic arsenic dimethylarsinic acid (DMA), the roots and shoots of RCc3p‐AEY transgenic plants had similar As accumulation compared to the WT, demonstrating the substrate specificity of ScYCF1 and OsABCC1 for with As (III) (Figure S7a, b). To verify that DMA is not a major substrate of OsABCC1 and ScYCF1, we performed DMA sensitivity tests using yeast of different genotypes of OsABCC1 and ScYCF1. There is no difference in DMA sensitivity between the *ycf1* null mutant and its isogenic wild type, or between SM7 expressing OsABCC1 and SM7 transformed with empty vector (Figure S7c). These results strongly suggest that the two transporters do not recognize DMA as their substrate and that the vacuolar compartmentalization of As in roots inhibits inorganic As translocation to the shoot (Figure 2a, b, c). There was no observable difference in Cd concentration and root‐to‐shoot translocation in any of the transgenic plants tested in this study (Figure S7d, e, f).

An immunohistochemistry analysis of the roots of the *RCc3p::GUS* plants revealed that the *RCc3* promoter mediated high expression of the gene construct in the cortical cell layer of the root, which constitutes most of the rice root volume, and the endodermis, where Lsi2 mediates the efflux of As and promotes its loading into the xylem (Ma *et al*., 2008) (Figure 1a). To determine whether As is specifically compartmentalized in the root cortical cells of RCc3p‐AEY, we performed several different assays. First, we compared the As concentrations in two root zones, one with live cortical cells (0–3 mm from the tip) and the other with aerenchyma (3–8 mm). Only the root segments of the RCc3p‐AEY plants containing live cortical cells accumulated more As than the WT (Figure S8a). Furthermore, the highest As concentrations were found in the RCc3p‐AEY root samples enriched with cortical layers, prepared using the stepwise grinding method described by Song *et al*. (2011) (Figure 2d, e). In addition, thiols, which are the major chelators of As and colocalize with As (Moore *et al*., 2011; Raab *et al*., 2004), were found to accumulate to high levels in the vacuoles of the root cortex cells in the elongation zone (2–3 mm from the root tip) of the RCc3p‐AEY plants, but not in the WT (Figures 2f, S8b). These results suggest that the vacuolar As compartmentalization in the root cortex, mediated by *OsABCC1* and *ScYCF1* concomitantly with γ*‐ECS*, was the major contributing factor to the reduced As translocation to the shoot and grains of the RCc3p‐AEY rice plants.

### Reduced As translocation from internode II to the grain of RCc3p‐AEY plants

**Figure 3:**
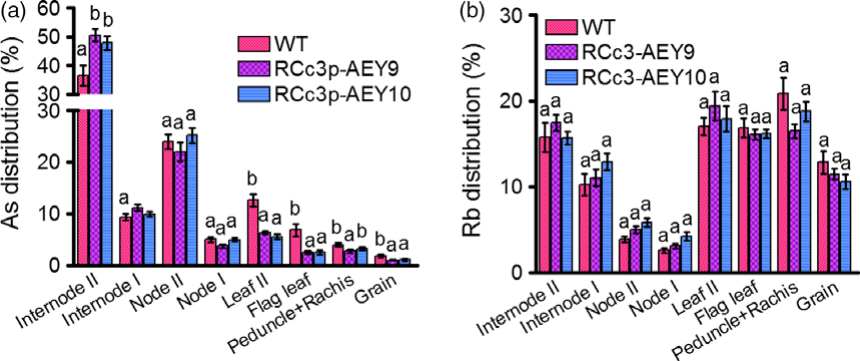
Reduced As translocation from internode II to the grain. WT and RCc3p‐AEY plants were cultured in 1/2 Kimura medium until the milk stage of grain filling. Plants were cut below internode II and then treated with half‐strength Kimura solution supplemented with 10 μm As(III) and 10 μm Rb(I) for 24 h. After incubation, each organ was harvested to measure As (a) and Rb (b). The values are means and standard errors (*n* = 7 plants). Different letters indicate significantly different means (Tukey's multiple comparison analysis, *P* ≤ 0.05).

The node is the most important tissue for As redistribution and transfer into the rice grain (Chen *et al*., 2015; Moore *et al*., 2014; Song *et al*., 2014). A defect in vacuolar As sequestration in phloem companion cells of node I was previously demonstrated to cause a 10‐fold increase in the As accumulation of rice grains (Song *et al*., 2014). Our immunohistochemistry results (Figure 1a) suggest that the two As transporters introduced into the transgenic rice might also function in the phloem cells of the node and internode. To determine whether the OsABCC1 and YCF1 vacuolar As transporters decreased the translocation of As to the grains from the nodes or the internodes in the RCc3p‐AEY transgenic plants, we performed As feeding assays using stems from upper internode II at the milk stage of grain filling. The RCc3p‐AEY transgenic lines exhibited a highly reduced As concentration in the leaf, flag leaf and grain (Figures 3a, S9a), and accumulated more As than the WT in internode II, which was directly exposed to the As(III) solution (Figure 3a); however, the As distribution was similar in the nodes of both the RCc3p‐AEY and WT lines. These results suggest that As compartmentalization in the nodes of the RCc3p‐AEY plants might not have contributed much to the inhibition of As translocation to the leaves and grains. Endogenous *OsABCC1* is likely already expressed in these tissues. The concentration and distribution of rubidium (Rb), the control metal ion used in the feeding assays, was similar in all plants (Figures 3b, S9b). Our results indicate that RCc3p‐AEY rice could efficiently reduce As accumulation in the rice grains by inhibiting the movement of As into the shoot. Furthermore, vacuolar sequestration of As in the root cortex and phloem cells of the internode is an effective strategy for reducing the accumulation of As in grains.

### Grain yield in RCc3p‐AEY transgenic plants

We examined the agronomic traits in RCc30‐AEY transgenic plants cultivated in rice paddy fields to clarify whether hyperaccumulation of As and thiols in the root reduces plant growth and grain yield. We compared the major agronomic traits of four different transgenic lines and WT plants, including plant height, tiller number, panicle length, 1000 grain weight and panicle weight. All of the transgenic lines resembled the WT with respect to these traits (Table 1).

**Table 1:** Agronomic traits in RCc3p‐AEY rice plants. The agronomic traits were examined in plants cultivated at paddy fields in 2017. The values are means and standard errors (*n* = 5 plants). The same letters indicate no significantly different means (Tukey's multiple comparison analysis, *P* ≤ 0.05)

Genotypes	Plant height (cm)	Tiller number	Panicle length (cm)	Grain weight (g/1000 grains)	Panicle weight (g/panicle)
WT	105.0 ± 1.060^a^	11.1 ± 0.167^a^	22.6 ± 0.447^a^	22.5 ± 0.639^a^	4.5 ± 0.128^a^
RCc3p‐AEY‐3	104.6 ± 1.037^a^	11.0 ± 0.728^a^	23.0 ± 0.354^a^	22.9 ± 0.761^a^	4.5 ± 0.152^a^
RCc3p‐AEY‐6	105.4 ± 1.525^a^	11.4 ± 0.909^a^	22.6 ± 0.671^a^	22.1 ± 0.564^a^	4.4 ± 0.113^a^
RCc3p‐AEY‐9	106.0 ± 1.275^a^	11.3 ± 1.085^a^	22.6 ± 0.570^a^	23.0 ± 0.715^a^	4.6 ± 0.143^a^
RCc3p‐AEY‐10	104.6 ± 0.908^a^	11.1 ± 0.890^a^	23.0 ± 0.790^a^	22.3 ± 0.604^a^	4.4 ± 1.121^a^

## Conclusion

**Figure 4:**
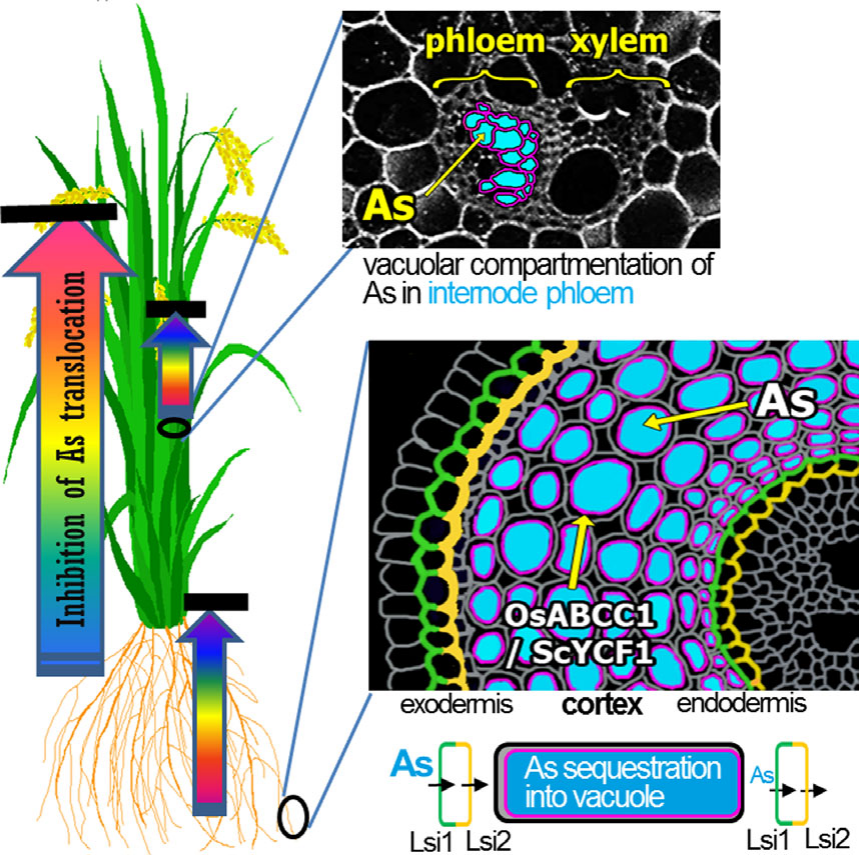
Vacuolar sequestration of As to reduce the amount of As in the grain. WT plants efficiently take up and translocate As into the xylem through the Lsi1 (green colour) and Lsi2 (yellow colour) transporters located in the root exodermis and endodermis. However, RCc3p‐AEY plants sequester As (blue colour) into cortical cell vacuoles via OsABCC1 and ScYCF1 (pink colour). This sequestration inhibits radial translocation of As into the endodermis, where As is translocated into the xylem via Lsi2 and decreases As translocation to the shoot. Furthermore, in phloem cells of the internode, OsABCC1 and ScYCF1 sequester As into vacuoles and decrease As translocation to the grain.

Rice is a staple food for billions of people in Asia, and its consumption is increasing in Europe (http://airea.net/images/siteimages/EU27-Rice.pdf) and the USA because it is regarded as a healthy, gluten‐free alternative source of carbohydrates (Batres‐Marquez *et al*., 2009). However, the As content of rice poses a serious problem (Schoof *et al*., 1999). Particularly alarming are baby foods based on rice, which were considered to be nutritious and safe, but in fact contain relatively high amounts of As and can cause diseases and developmental problems in children (Karagas *et al*., 2016). Therefore, the As content in rice grains is a critical issue for human health. Here, we successfully developed a strategy to reduce As in rice grains by enhancing As vacuolar sequestration through the transgenic expression of *OsABCC1*,* ScYCF1* and γ*‐glutamylcysteine synthetase*, while preserving grain yield. The two ABC transporters for As vacuolar compartmentation were driven by *OsRCc3p,* a tissue‐specific promoter driving expression in the root cortical cell layer, phloem cells of nodes and internodes. Root‐to‐shoot As translocation was greatly reduced in the transgenic plants due to the elevated vacuolar sequestration of As in root cortical cells. This sequestration probably inhibits the radial translocation of As from the cortex to the endodermis, where Lsi2, a major As efflux transporter localized to the proximal side of the endodermal layer, loads As into the xylem (Figure 4). Furthermore, the reduced shoot‐to‐grain translocation of As mediated by the transgenically expressed *OsABCC1* and *ScYCF1* in the phloem regions of nodes and internodes also contributed to the decreased As accumulation in grain (Figure 4). These results suggest that sequestering As into tissues involved in As translocation is an important step for developing low As rice. However, if these plants are grown in paddy fields containing excess As, their high vacuolar sequestration of As in nongrain tissues leads to hyperaccumulation and consequential cell damage, especially in the root. Therefore, we plan to improve our As‐safe rice by increasing the root's As extrusion activity into the rhizosphere.

## Experimental procedures

### Rice culture conditions

Rice seeds (*Oryza sativa* L.) were germinated on half‐strength Murashige and Skoog (1/2 MS) agar plates. After 10 days, the seedlings were transferred to normal soil with basal levels of As (2.8 ± 0.5 mg/kg dried soil; below the acceptable soil As limits in Korea (25 mg/kg)) or to half‐strength (1/2) Kimura B hydroponic medium (Yoshida, 1976) supplemented with 0.2 mm urea and cultured in a glasshouse at 25–35 °C under natural light or in a growth room at 28 °C under 16 h light/8 h dark conditions. To analyse As accumulation in the rice seedlings, the 4‐week‐old plants grown hydroponically in the growth room were treated with 1 μm As(III) and 0.1 μm Cd(II) for five days or 2 μm DMA(V) for two days. The shoots and roots were washed with ice‐cold water and harvested to measure their As and Cd contents. To investigate the As distribution at the reproductive stage, rice plants at the milk stage of grain development, which had been grown in the hydroponic medium in the glasshouse were treated with 1.0 μm As (III) for 10 days. Samples were prepared of the root, basal stem (1 cm), internodes, nodes, leaves, flag leaf, peduncle, rachis and brown rice to measure As and Cd contents.

### Molecular cloning and construct design

To express *OsABCC1*,* ScYCF1* and γ*‐ECS* genes, the rice *RCc3* promoter (*RCc3p*), *Zea mays ubiquitin* promoter (*ZmUBIp*), and rice *Actin2* promoters (*ACTIN2p*) were isolated using PCR. The rice *RCc3* promoter (Xu *et al*., 1995) was amplified with a primer set (RCc3p‐F, RCc3p‐R) and a genomic DNA template from *O. sativa* (cv. Donging), while *ZmUBIp* and *ACTIN2p* were isolated using PCR with gene‐specific primer sets (ZmUBIp‐F and ZmUBIp‐R for *ZmUBIp*, Act2p‐F and Act2p‐R for *ACTIN2p*), using the pIPKb002 and pIPKb003 vectors as templates (Himmelbach *et al*., 2007). Sequences of all primers used are presented in Table S1. All PCR products were cloned using the pGEM‐T Easy Vector (Promega), and their sequence fidelities were confirmed by sequencing. To increase the number of multicloning sites available in the final vectors, restriction enzyme recognition sites were removed from the promoter sequences using site‐directed mutagenesis with gene‐specific primer sets; primers for the removal of the *Sac*I and *PmaC*I sites in the *RCc3* promoter were RCc3p_SacI‐1F, RCc3p_SacI‐1R, RCc3p_SacI‐2F, RCc3p_SacI‐2R, RCc3p_PmaCI‐F and RCc3p_PmaCI‐R; primers the for removal of the *Sal*I, *Xho*I, *Sac*I and *EcoR*I sites in *ZmUBIp* were ZmUBIp_SalI‐F, ZmUBIp_Sal I‐R, ZmUBIp_XhoI‐F, ZmUBIp_XhoI‐R, ZmUBIp_SacI‐F, ZmUBIp_SacI‐R, ZmUBIp_EcoRI‐F and ZmUBIp_EcoRI‐R; primers for the removal of the *EcoR*I, *Sac*I and *BamH*I sites in *ACTIN2p* were Act2p_EcoRI‐F, Act2p_EcoRI‐R, Act2p_SacI‐F, Act2p_SacI‐R, Act2p_BamHI‐F and Act2p_BamHI‐R.

The modified *ZmUBI* promoter was subcloned into the *BstX*I and *EcoR*I sites of the pCAMBIA1300, pCAMBIA1302 and pBSK vectors (ZmUBIp1300, ZmUBIp1302, pBSK_ZmUBIp) or *BamH*I and *EcoR*I sites of the pBSK vector (pBSK_ZmUBIp‐1). The modified *RCc3* promoter was subcloned into the *BstX*I and *EcoR*I sites of the pCAMBIA1300, pCAMBIA1302 and pBSK vectors (RCc3p1300, RCc3p1302, pBSK_RCc3p). *ACTIN2p* was inserted into the *Nco*I and *Hind*III sites of the pBSK vector to generate pBSK_Actp. Nos and 35S terminators were isolated from the pCAMBIA1302 vector using PCR and sequence‐specific primer sets (Nos_ter‐SalI and Nos_ter‐EcoRV, Nos_ter‐XbaI and Nos_ter‐BamHI, 35S_ter‐F and 35S_ter‐R) and then subcloned into the pGEM‐T Easy Vector. The Nos terminator was inserted into the *Sal*I and *EcoR*V sites of the pBSK_RCc3p vector and pBSK_ZmUBIp, or the *Xba*I and *BamH*I sites of the pBSK_ZmUBIp‐1 vector (pBSK_ZmUBIp_Noster, pBSK_RCc3p_Noster, pBSK_ZmUBIp‐1_Noster). The 35S terminator was subcloned into the *Xba*I and *Pst*I sites of the pBSK_Actp vector (pBSK_Actp_35Ster).

Full‐length *OsABCC1‐V5* was prepared from the pGK‐OsABCC1 vector (Song *et al*., 2014), inserted into the *BamH*I and *Xba*I sites of ZmUBIp1300, ZmUBIp1302, RCc3p1300 and RCc3p1302 to produce *ZmUBIp*::*OsABCC1‐V5* (UBIp‐A) and *RCc3p*::*OsABCC1‐V5* (*RCc3p*‐A). *Escherichia coli* γ*‐ECS (*Zhu *et al*., 1999) was isolated from the wild‐type strain (K12) using PCR with a sequence‐specific primer set (γ‐ECS_HindIII‐F, γ‐ECS_SalI‐R) and subcloned into the pGEM‐T Easy Vector for sequencing. The confirmed γ*‐ECS* gene was inserted into the *Hind*III and *Sal*I sites of the pBSK_ZmUBIp‐1_Noster and pBSK_Actp_35Ster vectors (pBSK_ZmUBIp‐1_γ‐ECS_Noster and pBSK_Actp_γ‐ECS_35Ster). To combine the *OsABCC1‐V5* and γ*‐ECS* genes in a single vector, the fragments of the 35S terminator‐*ACTIN2p*‐γ‐ECS (*Sal*I and *Ecl136*II fragments from the pBSK_Actp_γ‐ECS_35Ster vector) and Nos terminator‐UBI promoter‐γ*‐ECS* (*Sal*I and *Ecl126*II from the pBSK_ZmUBIp‐1_γ‐ECS_Noster vector) were inserted into the *Sal*I and *Pml*I sites of the ZmUBIp1301ABCC1‐V5 and RCc3p1301ABCC1‐V5 vectors, and the RCc3p‐AE (*RCc3p*::*OsABCC1‐V5*,* ZmUBIp*::γ*‐ECS*) and UBIp‐AE (ZmUBIp::*OsABCC1‐V5*,* ACTIN2p*::γ*‐ECS*) vectors were constructed.

To express *ScYCF1* in rice, codon‐optimized *ScYCF1* (*YCF1*) was synthesized and cloned into the *EcoR*I/*Xho*I sites of a pGK yeast shuttle vector. *YCF1* was subcloned into the *BamH*I and *Xba*I sites of ZmUBIp1300 and RCc3p1300 to construct the *ZmUBIp*::*YCF1* and *RCc3p*::*YCF1* vectors or inserted into the *EcoR*I and *Xba*I sites of the pBSK_ZmUBIp‐1_Noster and pBSK_RCc3p‐1_Noster vectors to produce pBSK_ZmUBIp‐1_YCF1_Noster and pBSK_RCc3p‐1_YCF1_Noster.

To generate the RCc3p‐AEY (*RCc3p*::*OsABCC1‐V5*,* ZmUBIp*::γ*‐ECS*,* RCc3p*::*ScYCF1*) vector, the two fragments of Nos terminator‐ZmUBIp‐γ‐ECS (*Xba*I and *Sal*I) from the pBSK_ZmUBIp_γ‐ECS_Noster vector and Noster‐RCc3pYCF1 (*Sal*I and *Ecl*136 II) from pBSK_RCc3p‐1_YCF1_Noster were inserted into the *Xba*I and *Pml*I sites of the ZmUBIp1301ABCC1‐V5 vector. The UBIp‐AEY (ZmUBIp::*OsABCC1‐V5*,* ACTIN2p*::γ*‐ECS*, ZmUBIp::*ScYCF1*) vector was constructed by ligating three fragments; the 35S terminator‐*ACTIN2p*‐γ‐ECS (*Xba*I and *Sal*I) from pBSK_Actp_γ‐ECS_35Ster vector, Noster‐ZmUBI promoter‐*YCF1* (*Sal*I and *Ecl136*II) from pBSK_ZmUBIp‐1_YCF1_Noster vector and the ZmUBIp1301ABCC1‐V5 (*Xba*I and *Pml*I) vector.

The coding region of the β*‐Glucuronidase* (*GUS*) gene was amplified by PCR with the pBI121 vector as template and the primer set GUS_EcoRI‐F and GUS_XbaI‐R, and then subcloned into the pGEM‐T Easy Vector. This gene was then excised with *EcoR*I and *Xba*I and inserted into the *EcoR*I and *Xba*I sites of the RCc3p1300 and ZmUBIp1300 vectors, respectively, to generate *RCc3p*::*GUS* and *ZmUBIp*::*GUS*.

### Generation of transgenic rice and expression analysis

The Dongjin cultivar, a currently cultivated *japonica* rice variety, was used for transformation mediated by *Agrobacterium tumefaciens,* as described previously (Hiei *et al*., 1994; Jeon *et al*., 1999), with the following modifications. Four‐week‐old calli were generated from the scutellum and cocultivated with the *Agrobacterium tumefaciens* strain GV3101 carrying the binary vectors (RCc3p‐A, RCc3p‐AE, RCc3p‐AEY, UBIp‐A, UBIp‐AE, UBIp‐AEY, RCc3p‐GUS or UBIp‐GUS) on 2N6 medium containing acetosyringone for 2–3 days in darkness at 25 °C. The calli were then washed three times with sterile water containing 100 mg/L cefotaxime, incubated for one day in liquid 2N6 medium supplemented with 50 mg/L hygromycin B and 250 mg/L cefotaxime, washed with sterile water and then incubated at 27 °C on 2N6 solid medium containing 50 mg/L hygromycin B and 250 mg/L cefotaxime for 4–5 weeks. The hygromycin‐resistant calli were transferred onto a preregeneration medium (2N6‐BA) containing 50 mg/L hygromycin B and 250 mg/L cefotaxime, where they were grown for 10 days before being transferred to a regeneration medium containing 25 mg/L hygromycin B and 250 mg/L cefotaxime for 4–6 weeks. The regenerated plants were grown in a 1/2 Kimura B hydroponic medium for 2 weeks and then transferred to soil. The expression of *OsRCc3* was investigated in the roots and node I of WT with primer sets (RCc3‐RT‐F and RCc3‐RT‐R, OsActin‐F, and OsActin‐R). To confirm the insertion of the transgenic genes into the rice genome, a genomic PCR was performed using gene‐specific primer sets (mOsABCC1‐F and mOsABCC1‐R for exogenous *OsABCC1*; mYCF1‐F and mYCF1‐R for *YCF1*; γECS‐F and γ‐ECS‐R for γ*‐ECS*). To examine whether the transformed genes were successfully expressed, qRT‐PCR was performed using Thermal Cycler Dice Real Time System (TP‐800, Takara Co., Japan) with gene‐specific primer sets (mOsABCC1‐F, mOsABCC1‐R, mYCF1‐F, mYCF1‐R, γ‐ECS‐F, γ‐ECS‐R, OsActin‐F and OsActin‐R).

### Stem As feeding assays

To investigate As translocation from the internodes or nodes to the grains, As feeding assays were performed following a previously published method (Song *et al*., 2014). The plants of the WT and RCc3p‐AEY lines were grown hydroponically until 10 days after flowering (milk stage of grain development). The stems were cut below node II, and then, internode II was immersed in a 250‐mL flask containing 80 mL 1/2 Kimura medium supplemented with 10 μm As (III) and 10 μm Rb (I) as the control metal for 24 h. Each organ was harvested to measure its As content.

### Determination of As and Cd concentrations in the xylem sap

To analyse the translocation of As and Cd from the root to the shoot, 4‐week‐old hydroponically grown rice seedlings were treated with 5.0 μm As (III) and 0.2 μm Cd for 4 h. The shoot was decapitated with a razor blade, and the xylem sap was collected with a micropipette for 45 min. The samples of collected sap were diluted to an equal volume with 5% HNO_3_.

### Histochemical and immunohistochemical localization

To investigate the tissue‐specific *RCc3*‐ or *ZmUBI*‐driven expression of exogenous *OsABCC1* and *ScYCF1* in the transgenic plants, histochemical and immunohistochemical assays were performed in various tissues of *RCc3p*::*GUS* and *ZmUBIp*::*GUS* plants. Samples of the root, basal node, leaf sheath and leaf blade tissues were prepared from 2‐ to 3‐week‐old plants grown in a 1/2 Kimura B hydroponic medium, while samples of node I and the internode were collected from plants grown in soil during the grain‐filling stage. A GUS histochemical assay was performed as described previously (Jeon *et al*., 1999). After staining, the samples were dehydrated by sequentially soaking them in an ethanol series (50%, 70%, 90%, 95%, 100%). The hand‐sectioned samples were observed under a dissecting microscope (Olympus).

Immunostaining was performed using a GUS antibody (rabbit IgG antibody fraction; A‐5790, Molecular Probes), as described previously (Deng *et al*., 2013). Briefly, samples of the root, basal node, internode and node I were fixed using a fixation solution, sectioned to a 150‐μm thickness and treated with anti‐GUS. The fluorescence from the secondary antibody (Alexa Fluor 555 goat anti‐rabbit IgG; A21428, Molecular Probes) was observed using confocal laser‐scanning microscopy (Olympus FV‐1000 or Carl Zeiss LSM700).

### Thiol localization in the root

To determine thiol accumulation in the root, monobromobimane (mBB) staining was performed following the published method (Song *et al*., 2014). Roots were collected from 2‐ to 3‐week‐old plants treated with 1 μm As(III) for 3 h, incubated at room temperature in PBS buffer containing 15 μm mBB and 20 μm propidium iodide (PPI) for 30 min and then washed with PBS buffer. The fluorescence signal from mBB and PPI was observed using a confocal laser microscope (Olympus FV‐1000) and quantified using the ImageJ program (Abramoff *et al*., 2004).

### Immunoblot analysis

Microsome isolation and fractionation were performed according to a method described previously (Huang *et al*., 2016). The suspended microsomes were fractionated using discontinuous sucrose gradients (20%–60% sucrose in 10 mm Tris‐HCl, pH 7.6, 1 mm EDTA, and 1 mm DTT) by ultracentrifugation at 100 000 ***g*** for 18 h, which separated each membrane protein. After the fractionated membranes were recovered by a further ultracentrifugation at 100 000 ***g*** for 1 h, equal amounts of the samples (20 μg for the OsABCC1 and V5 detection; 5 μg for the detection of V‐ATPase, H^+^‐ATPase, and Bip) were incubated at 37 °C for 30 min. The samples were electrophoresed under the SDS‐PAGE gels (6% for OsABCC1 and ABCC1‐V5; 10% for H^+^‐ATPase, V‐ATPase and Bip) and transferred onto a nitrocellulose blotting membrane (GE Healthcare Life Sciences). The membranes were cross‐reacted with anti‐V5‐conjugated horseradish peroxidase (HRP) (R961‐25; Thermo Fisher Scientific; 5000‐times dilution), anti‐V‐ATPase (AS07213; Agrisera; 5000‐times dilution), anti‐H^+^‐ATPase (AS07260; Agrisera; 5000‐times dilution) and anti‐Bip (COP‐080017; Cosmo Bio; 5000‐times dilution). Anti‐rabbit antibody conjugated with HRP (Promega; 10 000‐times dilution) was used as a secondary antibody, and the ECL Plus Western Blotting Detection System (32209; Thermo Fisher Scientific) was used to detect the chemiluminescence under an Image Quant LAS 4000 (GE Healthcare Life Sciences).

### Assay of As accumulation pattern in root

To compare the As accumulation patterns in the roots of the wild‐type and RCc3p‐AEY lines, a stepwise grinding method using liquid nitrogen was employed to separate the epidermis, cortex and vascular cell layers, as previously described (Song *et al*., 2011). The samples were analysed for As concentration, protein quantification was performed to normalize As concentration, and a qRT‐PCR analysis was used to determine the source of the cell layers in the root. The epidermis‐specific gene *OsZIP1* was used as a marker to determine the cell types of the isolated samples (Ogo *et al*., 2014). The primers used to detect *OsZIP1* expression are listed in Table S1.

### As measurement

To analyse As accumulation in grains and other organs, dried tissues were digested with 2–4 mL 65% HNO_3_ at 150 °C for 10–15 h. The metal(loid) concentrations were determined using an inductively coupled plasma mass spectrometer (ICP‐MS; Perkin Elmer).

### Grain yield investigation

To examine the grain yield of the transgenic rice plants, four independent RCc3p‐AEY lines and WT plants were cultivated in rice paddy fields located at the Kyung‐Hee University from May to October 2017. At harvest, the grain yield‐related agronomic traits were analysed.

### Statistical analyses

To analyse the statistical significance of the data, two‐tailed Student's *t*‐tests (significance level of *P < 0.05 or **P < 0.01) were performed using Microsoft Excel, or a one‐way analysis of variance (ANOVA, significance level of *P* < 0.05) was performed using GraphPad Prism 4.

## Supporting information


**Figure S1** Promoter‐GUS assays in plants transformed with *RCc3* promoter::GUS (RCc3p::GUS) or *ZmUBI* promoter::GUS (UBIp::GUS).
**Figure S2** Promoter‐GUS assays in plants transformed with *ZmUBI* promoter::GUS (UBIp::GUS).
**Figure S3** Development of transgenic plants transformed with *RCc3*p‐A (*RCc3* pro::*OsABCC1‐V5*), RCc3p‐AE (*RCc3* pro::*OsABCC1‐V5, ZmUBI* pro::γ*‐ECS*), or RCc3p‐AEY (*RCc3* pro::*OsABCC1‐V5, ZmUBI* pro::γ*‐ECS, RCc3* pro::*ScYCF1*).
**Figure S4** Development of transgenic plants transformed with UBIp‐A (*ZmUBI* pro::*OsABCC1‐V5)*, UBIp‐AE (*ZmUBI* pro::*OsABCC1‐V5, OsActin2* pro::γ*‐ECS*), or UBIp‐AEY (*ZmUBI* pro::*OsABCC1‐V5, OsActin2* pro::γ*‐ECS*,* ZmUBI* pro::*ScYCF1*).
**Figure S5** Cd and As accumulation in brown rice and flag leaves from T3 transgenic plants grown in soil.
**Figure S6** As concentration in roots and shoots of transgenic rice seedlings.
**Figure S7** Phenotypic analysis of the transgenic rice seedlings and yeast
strains subjected to the DMA and Cd treatments.
**Figure S8** As accumulation and thiols contents in roots.
**Figure S9** Reduced As translocation to grains of RCc3p‐AEY plants.
**Table S1** Primers used in this study.
