# Supplementary material for: Engineering rice with lower grain arsenic
Source: Plant Biotechnol J. 2018 Mar 25;16(10):1691–9. doi: 10.1111/pbi.12905 (PMC6131421; doi:10.1111/pbi.12905)
Supplement: Supplementary file 1 — Figure S1 Promoter‐GUS assays in plants transformed with RCc3 promoter::GUS (RCc3p::GUS) or ZmUBI promoter::GUS (UBIp::GUS). Figure S2 Promoter‐GUS assays in plants transformed with ZmUBI promoter::GUS (UBIp::GUS). Figure S3 Development of transgenic plants transformed with RCc3p‐A (RCc3 pro::OsABCC1‐V5), RCc3p‐AE (RCc3 pro::OsABCC1‐V5, ZmUBI pro::γ‐ECS), or RCc3p‐AEY (RCc3 pro::OsABCC1‐V5, ZmUBI pro::γ‐ECS, RCc3 pro::ScYCF1). Figure S4 Development of transgenic plants transformed with UBIp‐A (ZmUBI pro::OsABCC1‐V5), UBIp‐AE (ZmUBI pro::OsABCC1‐V5, OsActin2 pro::γ‐ECS), or UBIp‐AEY (ZmUBI pro::OsABCC1‐V5, OsActin2 pro::γ‐ECS, ZmUBI pro::ScYCF1). Figure S5 Cd and As accumulation in brown rice and flag leaves from T3 transgenic plants grown in soil. Figure S6 As concentration in roots and shoots of transgenic rice seedlings. Figure S7 Phenotypic analysis of the transgenic rice seedlings and yeast strains subjected to the DMA and Cd treatments. Figure S8 As accumulation and thiols contents in roots. Figure S9 Reduced As translocation to grains of RCc3p‐AEY plants. Table S1 Primers used in this study. [file PBI-16-1691-s001.docx]

**Supplementary Figure legends**

**Supplementary figure 1. Promoter-GUS assays in plants transformed with *RCc3* promoter::GUS (RCc3p::GUS) or *ZmUBI* promoter::GUS (UBIp::GUS).** Segments of leaf blade (a, e, i), leaf sheath (b, f, j), basal stem (c, g, k), and root (d, h, l) at the vegetative growth stage were stained with X-gluc for 12 h. GUS signals from RCc3p-GUS plants were detected in the basal stem and root, while UBIp::GUS plants exhibited GUS signals in all tissues. Bar = 1 mm. (m) Expression of *OsRCc3* in the roots and node I of wild-type rice was examined by RT-PCR. Equivalent amounts of total RNA (-RT) were used as templates for the negative controls.

**Supplementary figure 2. Promoter-GUS assays in plants transformed with *ZmUBI* promoter::GUS (UBIp::GUS).** Cross-sections of the root, flag leaf sheath, internode II, and node I were treated with the GUS antibody to verify GUS localization in these rice tissues. Red and blue signals indicate GUS and cell wall autofluorescence, respectively. The immune staining results suggest that genes driven by the *ZmUBI* promoter are ubiquitously expressed in transformed rice plants. Bar = 100 μm.

**Supplementary figure 3. Development of transgenic plants transformed with *RCc3*p-A (*RCc3* pro::*OsABCC1-V5*), RCc3p-AE (*RCc3* pro::*OsABCC1-V5,* *ZmUBI* pro::*γ-ECS*), or RCc3p-AEY (*RCc3* pro::*OsABCC1-V5,* *ZmUBI* pro::*γ-ECS, RCc3* pro::*ScYCF1*).** (a) Genomic PCR analysis confirmed the presence of the transformed genes. (b) Quantitative RT-PCR analysis reveal that the introduced genes of 10 independent RCc3p-AEY lines were indeed expressed in rice roots. The values are means and standard errors (n = 3). (c) RT-PCR analysis showed that *OsABCC1* is expressed in node I of independent RCc3p-AEY lines. Equivalent amounts of RNA (-RT) were used as templates for the negative controls. (d) Immunoblot analysis using the V5 antibody confirmed the presence of the intact OsABCC1-V5 protein in the RCc3p-AEY rice plant roots.

**Supplementary figure 4. Development of transgenic plants transformed with UBIp-A (*ZmUBI* pro::*OsABCC1-V5)*, UBIp-AE (*ZmUBI* pro::*OsABCC1-V5,* *OsActin2* pro::*γ-ECS*), or UBIp-AEY (*ZmUBI* pro::*OsABCC1-V5,* *OsActin2* pro::*γ-ECS*, *ZmUBI* pro::*ScYCF1*).** (a) Maps of vectors carrying UBIp-A, UBIp-AE, and UBIp-AEY. (b) Genomic PCR analysis confirmed the presence of the transformed genes. (c) Quantitative RT-PCR analysis revealed that the introduced genes were expressed in rice roots. The values are means and standard errors (n = 3 replicates). (d) Immunoblot analysis using the V5 antibody confirmed the presence of the intact OsABCC1-V5 protein in the rice UBIp-AEY plant roots.

**Supplementary figure 5. Cd and As accumulation in brown rice and flag leaves from T3 transgenic plants grown in soil.** Contents of Cd (a, b) and As (c) were measured in the grains (a) and flag leaves (b, c) from WT and transgenic plants (RCc3p-A, RCc3p-AE, RCc3p-AEY, UBIp-A, UBIp-AE, and UBIp-AEY). The values are means and standard errors (n = 5 plants). The different letters indicate significantly different means (Tukey’s multiple comparison analysis, *P* ≤ 0.05).

**Supplementary figure 6. As concentration in roots and shoots of transgenic rice seedlings.** WT and transgenic plants (RCc3p-A, RCc3p-AE, RCc3p-AEY, UBIp-A, UBIp-AE, and UBIp-AEY) were cultured in hydroponic medium for four weeks. As contents were measured in the roots (a) and shoots (b) of plants treated with 1 μM As(III) for five days (n = 8). The values are means and standard errors. The different letters indicate significantly different means (Tukey’s multiple comparison analysis, *P* ≤ 0.05).

**Supplementary figure 7.** Phenotypic analysis of transgenic rice seedlings and yeast strains grown in the presence of DMA and Cd. WT and transgenic plants (RCc3p-A, RCc3p-AE, RCc3p-AEY, UBIp-A, UBIp-AE, and UBIp-AEY) were cultured in hydroponic medium for four weeks. DMA (a, b) and Cd (d, e) contents were measured in the roots (a, d) and shoots (b, e) of plants treated with 2 μM DMA (a, b) or 0.1 μM Cd(II) (e, f) for two or five days (a and b, n = 5; e and f, n = 8). To determine whether ScYCF1 and OsABCC1 transport DMA, the DMA sensitivity of the *ycf1* yeast mutant and SM7 yeast expressing OsABCC1 were compared with their respective control yeast strains (*ycf1* *vs.* WT, *OsABCC1* *vs.* EV) (c). To analyze the translocation of Cd (f), xylem sap was collected from plants treated with 0.2 μM Cd(II) for 4 h (n = 5 plants). The values are means and standard errors. The different letters indicate significantly different means (Tukey’s multiple comparison analysis, *P* ≤ 0.05).

**Supplementary figure 8. As accumulation and thiols contents in roots.** (a) As accumulation pattern in the roots of WT and RCc3p-AEY plants. As contents were measured in root segments of three-week-old plants treated with 1 μM As(III) for one day (n = 5 batches of 50 root segments). (b) Accumulation of thiols in roots of WT and RCc3p-AEY plants. Root segments (2–3 mm from the tip) were prepared from three-week-old plants treated with 1 μM As(III) for 3 h and stained with 15 μM monobromobimane for 30 min. The intensity of the blue fluorescence was measured using Image J (N4 roots, n = 16 root cells). The different letters indicate significantly different means (Tukey’s multiple comparison analysis, *P* ≤ 0.05).

**Supplementary figure 9. Reduced As translocation to grains of RCc3p-AEY plants.** WT and RCc3p-AEY plants were cultured in 1/2 kimura medium until the milk stage of grain filling. Plants were cut below internode II and then treated with half-strength Kimura solution supplemented with 10 μM As(III) and 10 μM Rb(I) for 24 h. After incubation, each organ was harvested to measure As (a) and Rb (b). The values are means and standard errors (n = 5 plants). The different letters indicate significantly different means (Tukey’s multiple comparison analysis, *P* ≤ 0.05).

**Supplementary Figures**


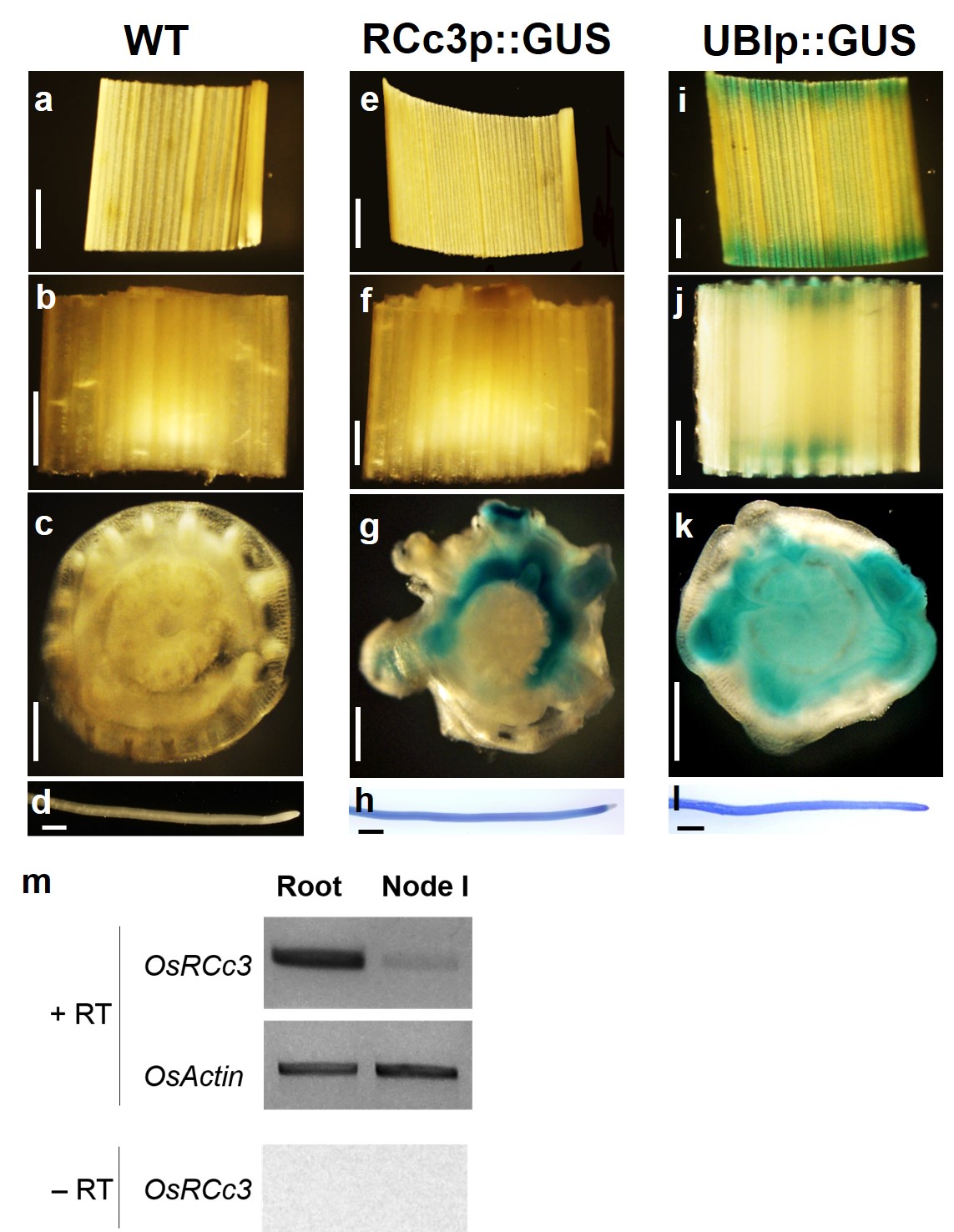
**Supplementary figure 1. Promoter-GUS assays in plants transformed with *RCc3* promoter::GUS (RCc3p::GUS) or *ZmUBI* promoter::GUS (UBIp::GUS).**


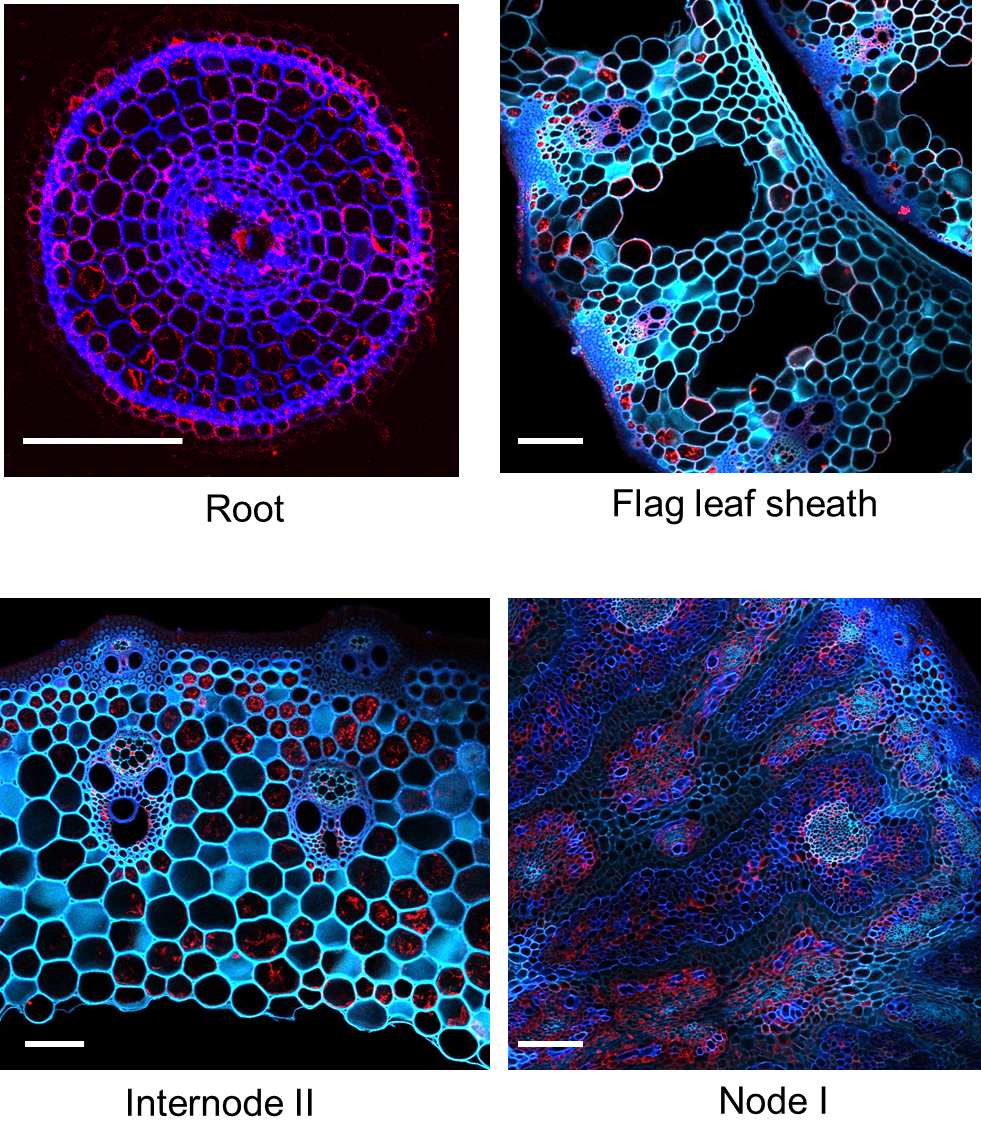


**Supplementary figure 2. Promoter-GUS assays in plants transformed with *ZmUBI* promoter::GUS (UBIp::GUS).**


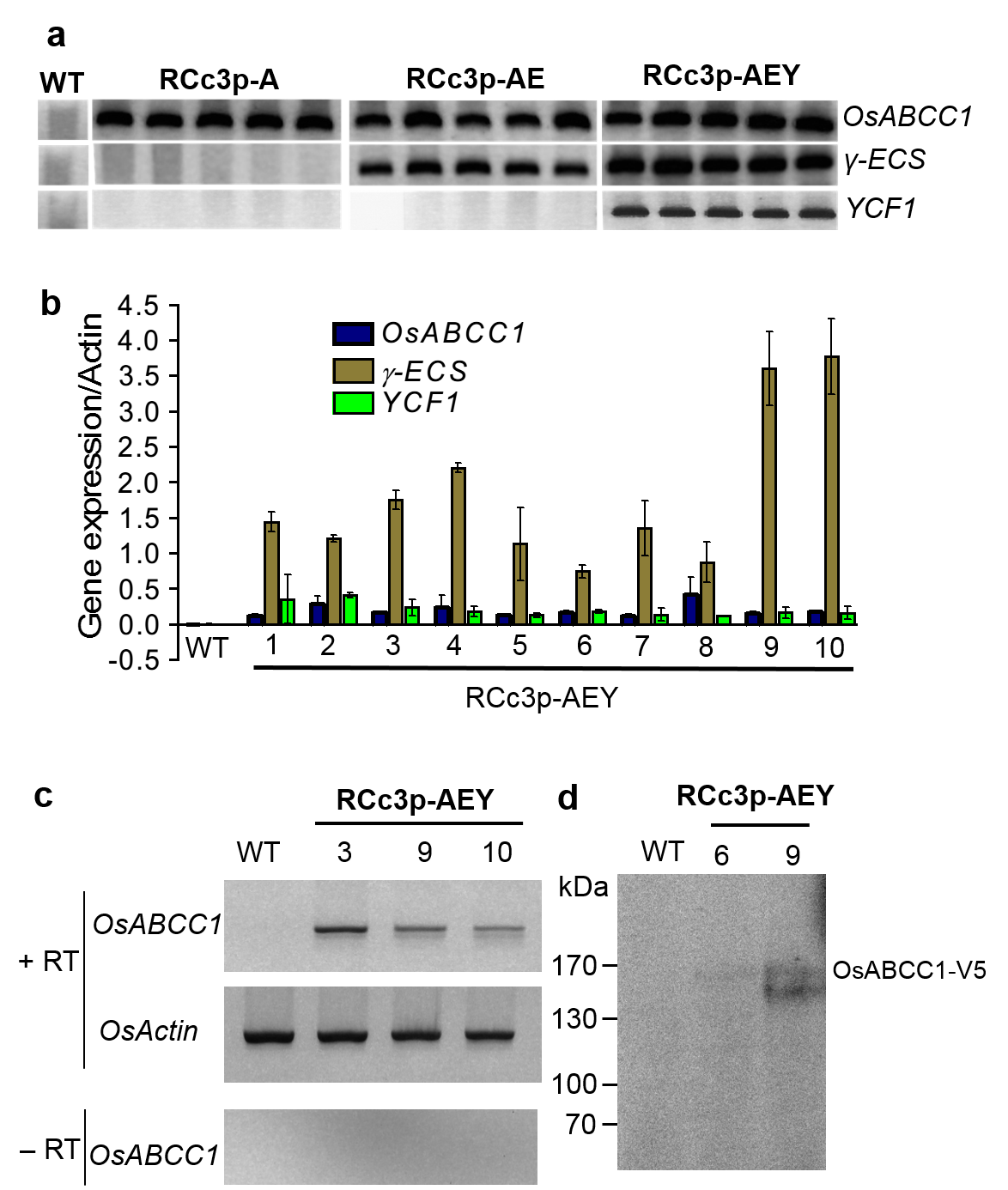


**Supplementary figure 3. Development of transgenic plants transformed with *RCc3*p-A (*RCc3* pro::*OsABCC1-V5*), RCc3p-AE (*RCc3* pro::*OsABCC1-V5,* *ZmUBI* pro::*γ-ECS*), or RCc3p-AEY (*RCc3* pro::*OsABCC1-V5,* *ZmUBI* pro::*γ-ECS, RCc3* pro::*ScYCF1*).**


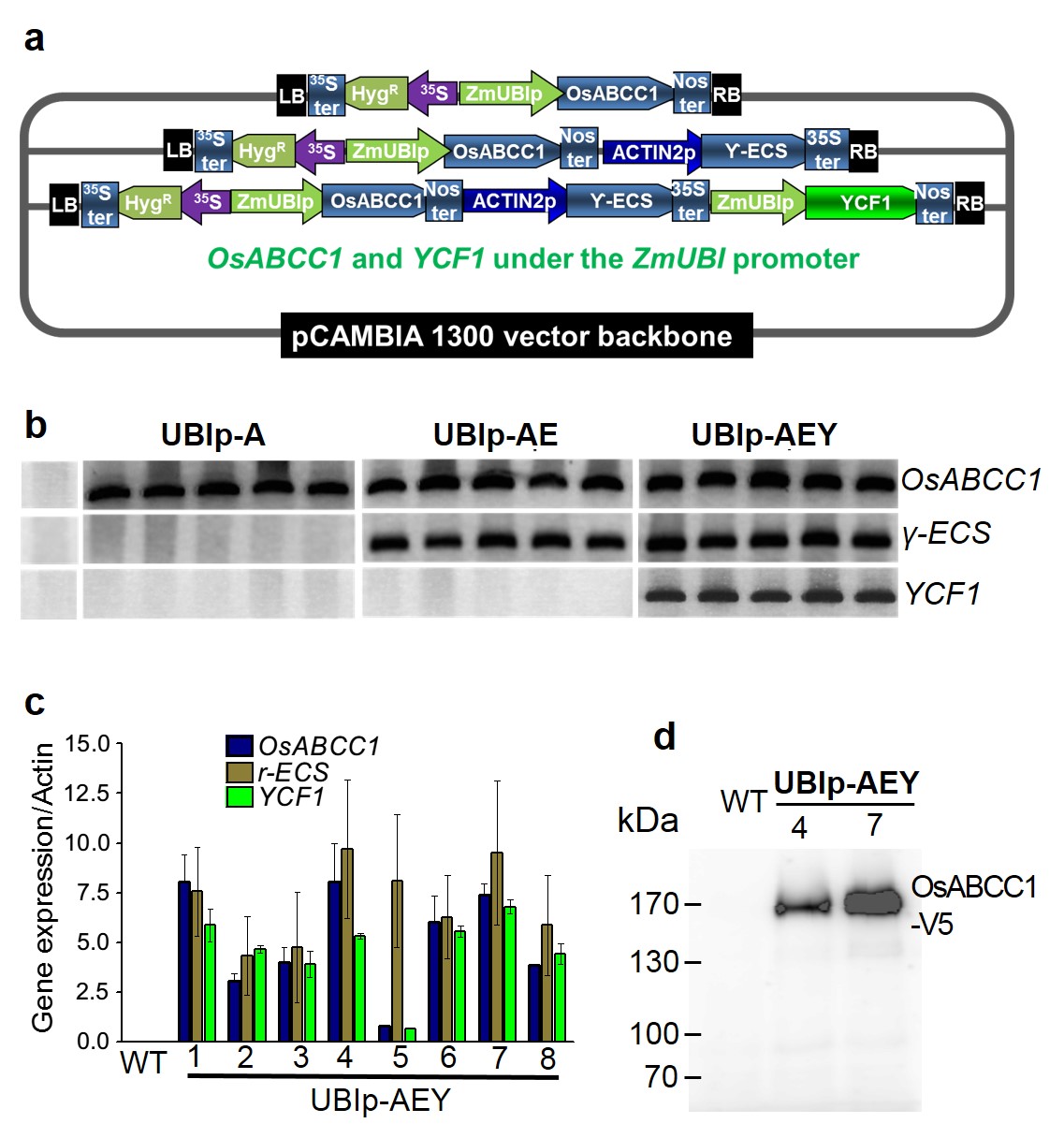


**Supplementary figure 4. Development of transgenic plants transformed with UBIp-A (*ZmUBI* pro::*OsABCC1-V5)*, UBIp-AE (*ZmUBI* pro::*OsABCC1-V5,* *OsActin2* pro::*γ-ECS*), or UBIp-AEY (*ZmUBI* pro::*OsABCC1-V5,* *OsActin2* pro::*γ-ECS*, *ZmUBI* pro::*ScYCF1*).**


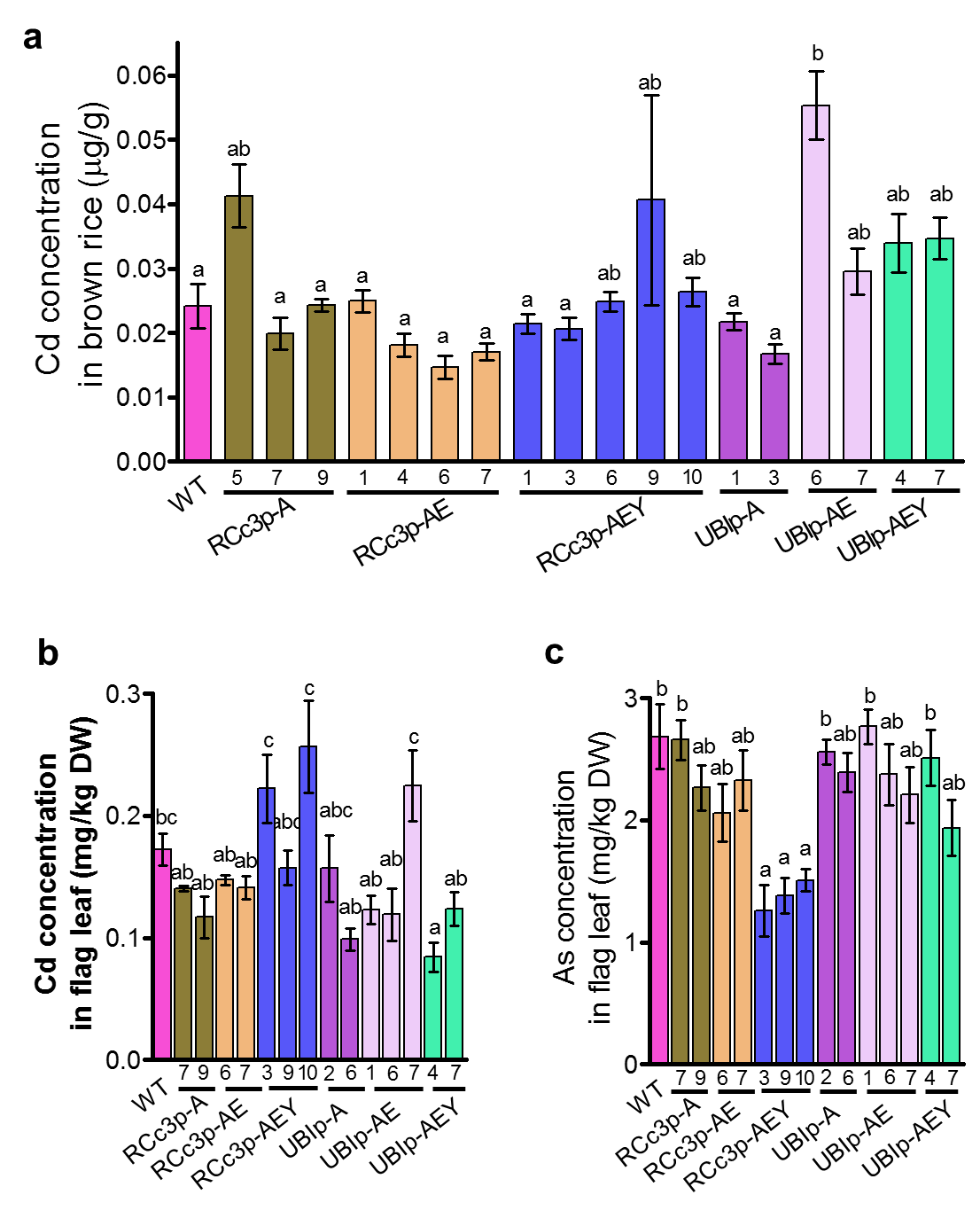


**Supplementary figure 5. Cd and As accumulation in brown rice and flag leaves from T3 transgenic plants grown in soil.**


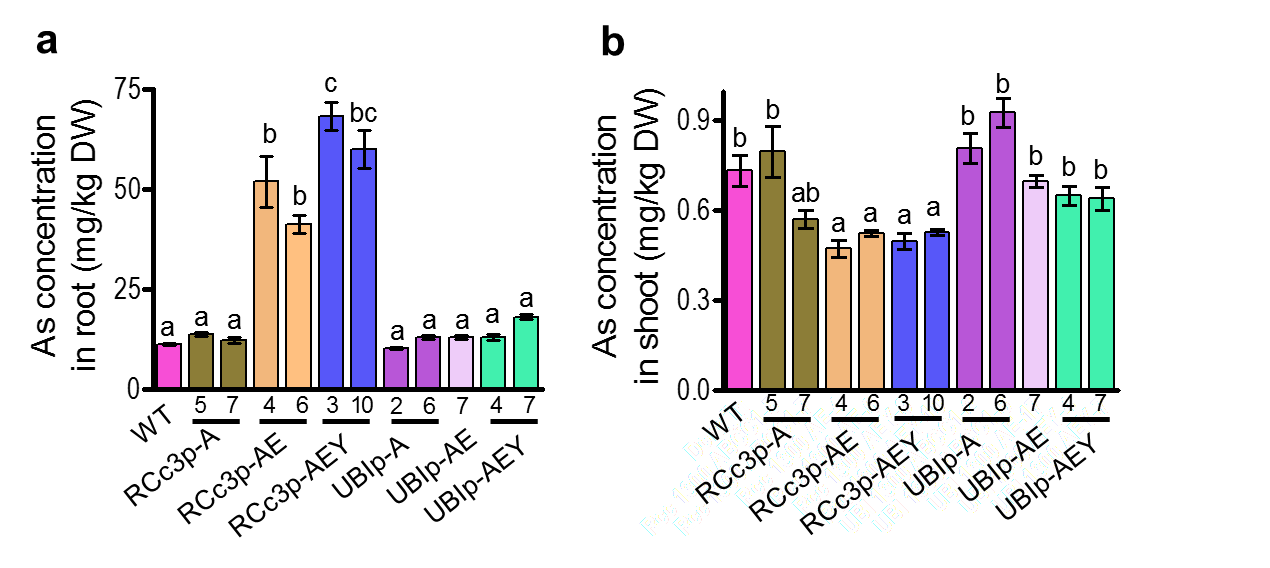


**Supplementary figure 6. As concentration in roots and shoots of transgenic rice seedlings.**


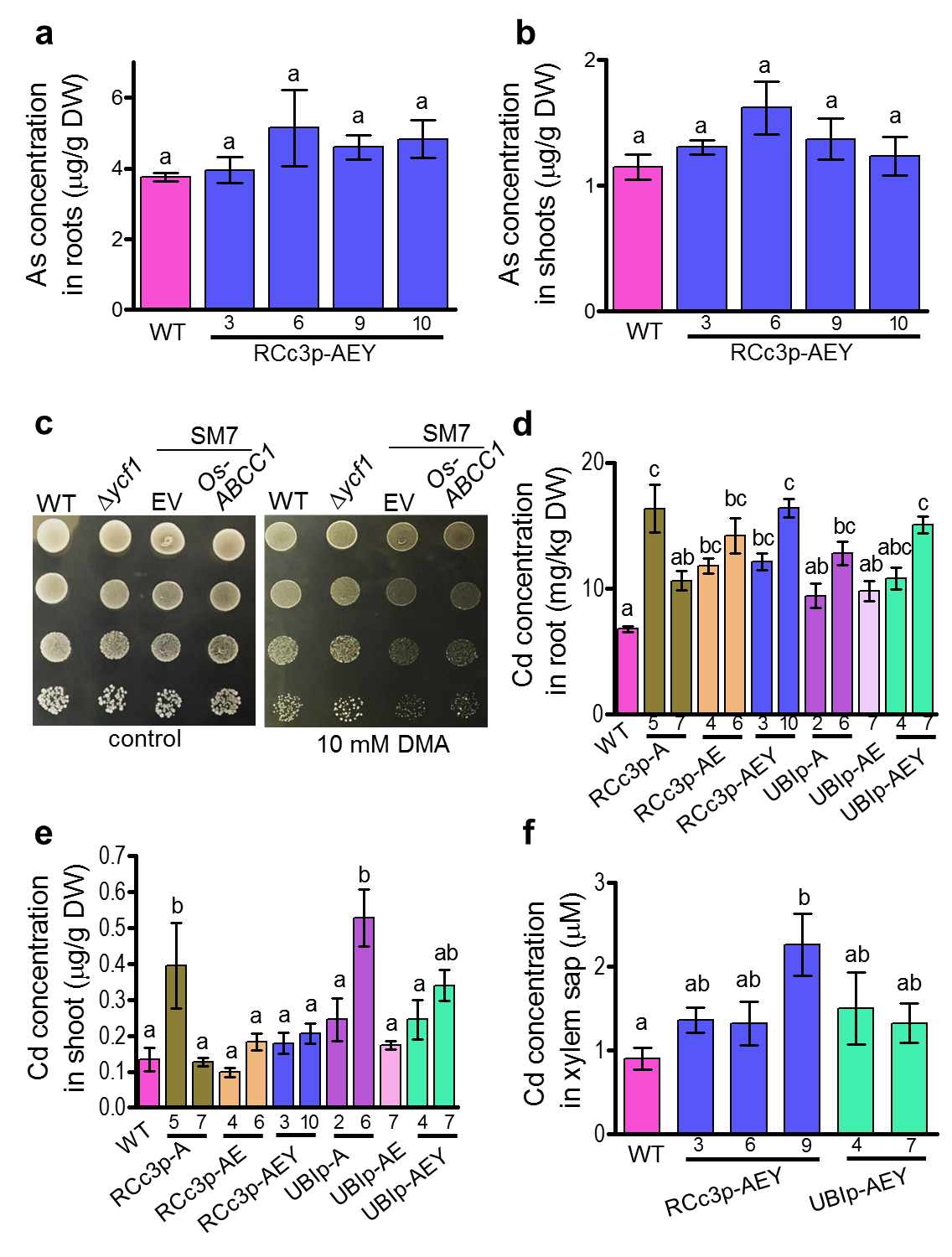


**Supplementary figure 7.** Phenotypic analysis of transgenic rice seedlings and yeast strains under the DMA and Cd conditions.


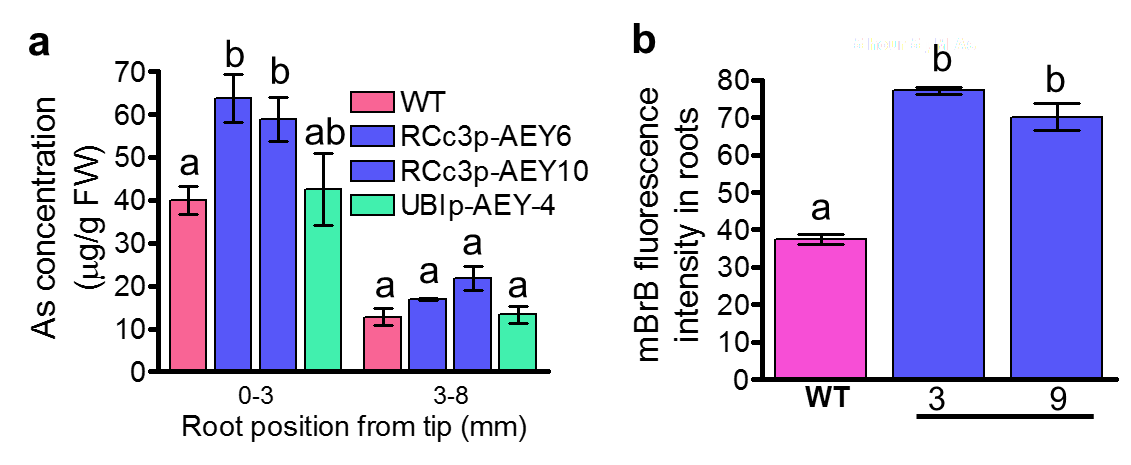


**Supplementary figure 8. As accumulation and thiols contents in roots.**


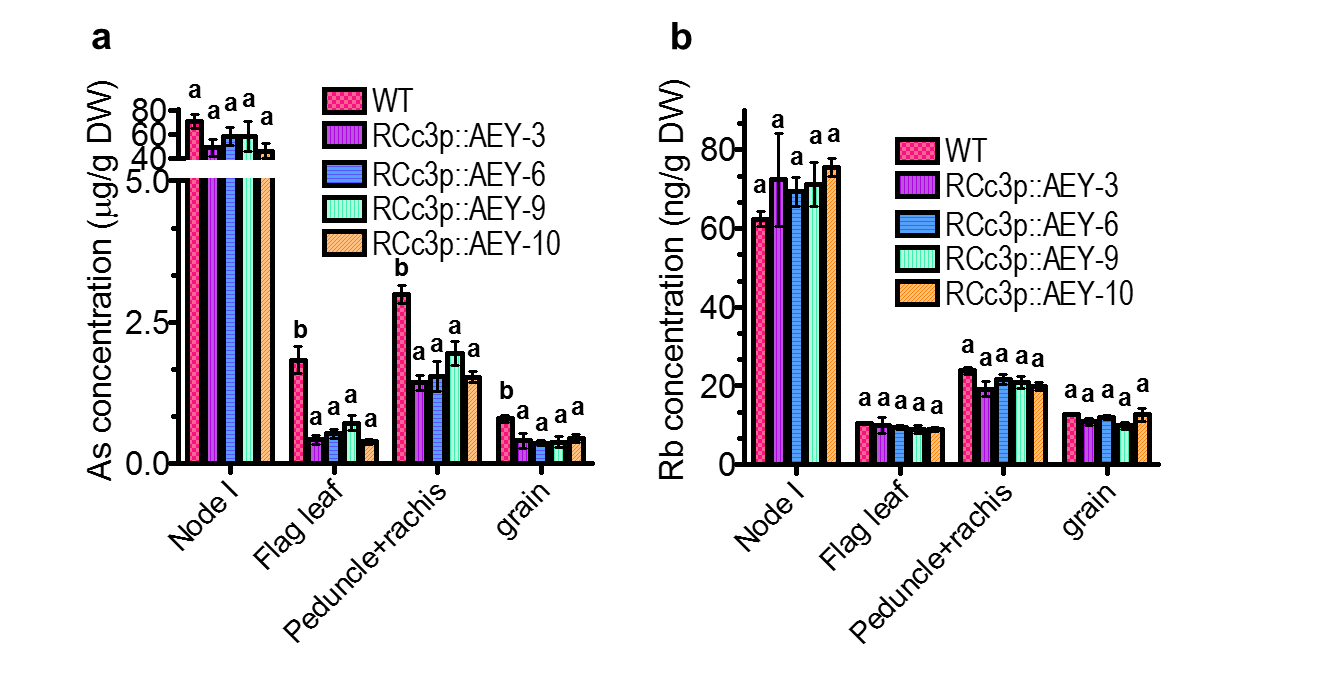


**Supplementary figure 9. Reduced As translocation to grains of RCc3p-AEY plants.**

**Supplementary Table 1. Primers used in this study.**

| Primer name | Primer sequence (5′–3′) |
| --- | --- |
| RCc3p-F | AACAAATAAGACCACCATGTTGGCAACAAGCCTCGACGC |
| RCc3p-R | CTACGTACCCGAATTCGATCGATCACAAGCGCAG |
| RCc3p_EcoRV-F | GATATCCCACCATGTTGGCAACAAGCCTC |
| ZmUBIp-F | GGATCCCCACCATGTTGGAGATAATGAGCATTGCATGTCTAAG |
| ZmUBIp-R | GAATTCAAGTAACACCAAACAACAGG |
| Act2p-F | GGATCCCCACCATGTTGGCACGAGGTCATTCATATGC |
| Act2p-R | GAATTCTACAAAAAAGCTCCGCACGAG |
| RCc3p_SacI-1F | GGGTTCACATGAGCTGTAACG |
| RCc3p_SacI-1R | CGTTACAGCTCATGTGAACCC |
| RCc3p_SacI-2F | CTGATCAAGAGCACTTAATTAGC |
| RCc3p_SacI-2R | GCTAATTAAGTGCTCTTGATCAG |
| RCc3p_PmacI-F | CCTAGTAGCCAAGTGCCTAG |
| RCc3p_PmacI-R | CTAGGCACTTGGCTACTAGG |
| UBIp_SalI-F | AAACGCCGTTGACGAGTCTAA |
| ZmUBIp_Sal I-R | TTAGACTCGTCAACGGCGTTT |
| ZmUBIp_XhoI-F | ACCCCTGTCGAGAGTTCCGC |
| ZmUBIp_XhoI-R | GCGGAACTCTCGAGAGGGGT |
| ZmUBIp_SacI-F | GTTGTTCGGAGCGCACACACAC |
| ZmUBIp_SacI-R | GTGTGTGTGCGCTCCGAACAAC |
| ZmUBIp_EcoRI-F | TCGGAGTACAATTCTGTTTC |
| ZmUBIp_EcoRI-R | GAAACAGAATTGTACTCCGA |
| Act2p_EcoRI-F | TCAGGCGTATTCCACAATGAAC |
| Act2p_EcoRI-R | GTTCATTGTGGAATACGCCTGA |
| Act2p_SacI-F | CCGGACGACGAGGTCCTCCCC |
| Act2p_SacI-R | GGGGAGGACCTCGTCGTCCGG |
| Act2p_BamHI-F | TCGGCCCGGATCGTCGCGGGG |
| Act2p_BamHI-R | CCCCGCGACGATCCGGGCCGA |
| Nos_ter-Sal I | GTCGACGCTCGAATTTCCCCGATCGTTC |
| Nos_ter- EcoR V | GATATCTCCCGATCTAGTAACATAGATG |
| Nos_ter-Xba I | TCTAGAGCTCGAATTTCCCCGATCGTTC |
| Nos_ter-BamH I | GGATCCCGATCTAGTAACATAGATG |
| 35S_ter-F | TCTAGAACCGGAGTCCGCAAAAATCAC |
| 35S_ter-R | GGATCCAACGCTCGACGCGGCCGCC |
| γ-ECS_HindIII-F | AAGCTTATGGCAATCCCGGACGTATCACAGGC |
| γ-ECS_SalI-R | GTCGACTCAGGCGTGTTTTTCCAGCCACAC |
| GUS_EcoRI-F | GAATTCATGTTACGTCCTGTAGAAACCCC |
| GUS_XbaI-R | TCTAGATTGTTTGCCTCCCTGCTGCGGTTTTTC |
| mOsABCC1-F | TCCTGAACCTGCTGCTGGAAAGC |
| mOsABCC1-R | TTTCGGCATCGGTGCTGATCAGG |
| mYCF1-F | ATTGACAACATCGCCATCAACGAG |
| mYCF1-R | TCGAGAACGATGATACGATCGCTG |
| γECS-F | ATGGCAATCCCGGACGTATCACAG |
| γECS-R | TTCGCTTGCCAGAATGCCATTGGC |
| OsActin_RT-F | TCCATCTTGGCATCTCTCAG |
| OsActin_RT-R | GTACCCGCATCAGGCATCTG |
| OsZIP1-F | CTCTTCAAGTTCCTCGCCGTCCTCCT |
| OsZIP1-R | CGGCCACGATTAATGAATGGGGTG |
| RCc3-RT-F | ATGCCTGCATGGGTTGATCCT |
| RCc3-RT-R | CGTGATACGTACAAACAGCCAGCAT |
